# Advances in Radiation Oncology for the Treatment of Cervical Cancer

**DOI:** 10.3390/curroncol29020079

**Published:** 2022-02-09

**Authors:** Mame Daro Faye, Joanne Alfieri

**Affiliations:** Division of Radiation Oncology, McGill University Health Centre, Montreal, QC H4A 3J1, Canada; mame.faye@mail.mcgill.ca

**Keywords:** cervical cancer, radiation oncology, radiotherapy, chemoradiation, IMRT, brachytherapy, 3D-IGABT, SBRT, immunotherapy, immune checkpoint inhibitors

## Abstract

Background: Over the past two decades, there has been significant advancement in the management of cervical cancer, particularly in the domain of definitive chemoradiotherapy for locally advanced cervical cancer (LACC). Indeed, radiation treatment paradigms have shifted from a two-dimensional (2D) approach solely based on anatomical bony landmarks, to an image-guided three-dimensional (3D) approach, with the goal of delivering doses more precisely to clinical targets with an increased sparing of organs-at-risk. Methods: This is a narrative review on the advances in radiation technologies for the treatment of cervical cancer. Using the PubMed database, we identified articles published in English up until November 18, 2021 on the treatment of LACC with external beam radiotherapy (EBRT) and brachytherapy. A search of the Clinicaltrials.gov and Clinicaltrialsregister.eu retrieved information on ongoing clinical trials on the topic of combined immunotherapy and radiotherapy in cervical cancer. Results: We highlight the historical evolution from the use of 2D radiotherapy to 3D-conformal radiotherapy, and then intensity modulated radiotherapy (IMRT) for the delivery of EBRT. We also discuss advances in brachytherapy, notably the transition to 3D image-guided adaptive brachytherapy (3D-IGABT). In this context, we highlight large cohort studies that were recently constructed and have shown significant improvement in local control and treatment-related toxicities with 3D-IGABT. Finally, we discuss other advances in the field, notably the use of stereotactic body radiotherapy (SBRT) as a substitute to brachytherapy, and the addition of immunotherapy to chemoradiation. Conclusions: The use of IG-IMRT and 3D-IGABT have considerably improved treatment outcomes and toxicity profiles for patients with LACC, and are now considered the gold standard in many countries. The use of SBRT boost as a replacement for brachytherapy has been associated with increased toxicity and decreased efficacy and should be used with caution in the context of clinical trials. New experimental approaches include the addition of immunotherapy to chemoradiation regimens.

## 1. Introduction

Cervical cancer is the fourth most common cancer in women worldwide [1,2]. The majority of cervical cancers in developed countries are diagnosed early at stage I and 5-year overall survival (OS) for all stages remains above 73% [3,4]. However, outcomes for those with locally advanced cervical cancer remain quite poor. The 5-year OS for patients with regional disease is around 55% [3,5]. The addition of concurrent chemotherapy to radiotherapy (RT) has improved the prognosis of these patients; however treatment-related toxicity and distant recurrence remain a challenge [6]. Thus, there is much room for improvement in the treatment of locally advanced cervical cancer and new strategies are needed to further improve outcomes.

Standard treatment of locally advanced cervical cancer (LACC) consists of concurrent chemoradiotherapy (CCRT) with external beam radiotherapy (EBRT), followed by brachytherapy (BT) [7]. CCRT has been the standard of care for LACC since 1999, based on the results of five Phase III randomized controlled trials (RCT) showing a 30% to 50% survival advantage by adding cisplatin-based chemotherapy to radiation (GOG 85, GOG 120, GOG 123, SWOG 8797/Intergroup 0107, RTOG 9001) [8,9,10,11,12]. In the past two decades, radiation treatment paradigms have shifted from a two-dimensional (2D) approach, solely based on anatomical bony landmarks, to an image-guided three-dimensional (3D) approach, taking into account variations in tumour size and position, with the goal of delivering doses more precisely to clinical targets with an increased sparing of organs-at-risk (OARs). 

In this narrative review, we discuss the advances in external beam radiotherapy (EBRT) and brachytherapy (BT) for the treatment of locally advanced cervical cancer. We highlight the historical evolution from the use of 2D radiotherapy to 3D-conformal radiotherapy (3DCRT), and then to intensity modulated radiotherapy (IMRT) that has now become a standard for the planning and delivery of EBRT. We also discuss advances in brachytherapy, notably the evolution from a Manchester system delivery of BT, to now widespread use of 3D image-guided adaptive brachytherapy (3D-IGABT). In this context, we highlight recently conducted large cohort studies that have shown significant improvement in local control (LC) and treatment-related toxicities with 3D-IGABT. Finally, we highlight other technological advances in the field, notably the use of stereotactic body radiotherapy (SBRT) as a potential substitute when brachytherapy is contraindicated, and future directions such as the addition of immunotherapy to chemoradiation. 

## 2. Material and Methods

A literature search was performed in the PubMed database for articles on radiotherapy for the treatment of LACC. The following keywords were used in various search algorithms: “cervical cancer”, “radiotherapy,” “radiation therapy,” “chemoradiation”, “IMRT”, “brachytherapy”, “SBRT”, “immunotherapy,” and “immune checkpoint inhibitors”. Original research, review papers, or meeting abstracts published on the topic up to 18 November 2021 were considered. Articles published in languages other than English were excluded. Further references found within the articles and relevant to the subject were also used. A search query was also performed in Clinicaltrials.gov and Clinicaltrialsregister.eu to retrieve information on ongoing clinical trials on combined immunotherapy and RT in cervical cancer.

### 2.1. Advances in External Beam Radiotherapy

#### 2.1.1. From 2D Radiotherapy to 3D-Conformal Radiotherapy (3DCRT)

Historically, the treatment of LACC with RT has been performed with 2D external beam radiotherapy, solely based on empirically defined anatomical landmarks. These anatomical bony landmarks were defined based on X-rays to be able to largely cover the primary disease as well as any potential extent of the tumour to the adjacent soft tissues and draining lymph nodes. The most rudimentary technique of EBRT consists of two parallel opposed fields (AP-PA). This was followed by the use of Biaxial Telecobalt pendular irradiation of the pelvic lymph nodes combined with ^226^Radium-BT to treat cervical cancer in the 1960s, especially in the recurrent setting [13]. Later, the “four-field box” technique was introduced and achieved better OARs sparing. It consists of four large treatment fields, with the anterior border placed anteriorly to the pubic symphysis, posterior border covering the sacrum at the S3/S4 level, superior border at the level of the aortic bifurcation around the L3-L5 vertebral body levels, or at the level of T12 in the case of extended fields, when the para-aortic lymph nodes are involved. The inferior border is defined as the bottom of the obturator foramen and finally the lateral borders are placed 1.5 to 2 cm lateral to the pelvic brim. Although easily applicable, the use of standardized fields based only on bony landmarks has the caveat of not being adaptable to variations in individual patients’ anatomy and has been associated with geographical misses leading to decreased LC [14,15]. 

The computerized tomography (CT) scanner was invented in 1971 by Hounsfield and Cormack, but it was not until the 1990s that it was used in the planning and delivery of RT [16], marking the evolution from 2D-RT to 3DCRT. Indeed, 3DCRT takes advantage of the soft tissue and anatomical information obtained from a CT scan of the patient in a reproducible treatment position, to delineate the target disease and the neighboring OARs. Based on this information, gross tumour volume (GTV), clinical target volume (CTV) and planning target volume (PTV) are delineated as defined by the ICRU 50 and 62 reports [17,18]. This allowed for the use of the “four field box” technique based on anatomical rather than empirical bony landmarks, and the use of field blocks or multileaf collimators (MLCs) to better shape the dose distribution to the PTV while limiting the dose delivered to the OARs [19,20]. Compared to a 2D technique, 3DCRT also provides the advantage of recording volumetric dosimetry, correlating it with treatment outcomes and toxicities. 

#### 2.1.2. Intensity Modulated Radiotherapy (IMRT) for the Treatment of Locally Advanced Cervical Cancer

IMRT is a radiotherapy technique that allows for the delivery of highly conformal dose distribution compared to conventional techniques of 2D or 3DCRT, while minimizing the dose to nearby OARs. It achieves this through the use of multiple static beamlets or of volumetric intensity-modulated arcs (VMAT) and inverse planning software that can optimize the dose distribution, based on set constraints and target priorities. The trade-off is an increased volume of normal tissues receiving low doses of radiation. The success of IMRT at achieving such highly conformal dose distributions relies on the accuracy of target volume delineation and the quality of the imaging used. Thus, simulation CT scans are often fused with available diagnostic CT, MRI and PET/CT imaging to aid accurate target and OAR delineation. When available, MRI and/or PET/CT simulation is also favored to capture the anatomy in the treatment position. IMRT also allows for dose escalation to grossly involved pelvic and para-aortic lymph nodes by sequential or simultaneous integrated boost techniques when the planning CT is fused with diagnostic images such as a PET scan [21]. Multiple consensus contouring guidelines exist to help in the delineation of targets and OARs for cervical cancer [22,23] and have been extensively reviewed elsewhere [24].

Initial reports of the use of IMRT for the treatment of gynecological cancers were published in the early 2000s. In 2001, Portelance et al. showed that normal tissue sparing is superior with IMRT in a cohort of 10 patients with cervical cancer planned with IMRT vs. conventional techniques [25]. They showed that the volumes of bladder, rectum and small bowel receiving the prescribed dose of 45 Gy or higher was significantly decreased when using IMRT compared to conventional RT [25]. In 2002, Mundt et al. [26] reported their experience with intensity-modulated whole pelvic radiotherapy (IM-WPRT) in 40 women with gynecologic malignancies. They showed adequate coverage of the target volumes compared to conventional RT, with 98.1% of the PTV receiving the prescription dose. There was also an improved toxicity profile, with a decrease in grade 2 acute gastrointestinal toxicity in the IMRT group compared to the conventional whole pelvis RT group (60 vs. 91%, *p* = 0.002), and no patient developing grade 3 toxicity [26]. Since then, several other studies have shown a decrease in acute toxicities, notably of gastrointestinal (GI), genitourinary (GU) and hematological toxicities when using IMRT in the treatment of LACC compared to 2D or 3DCRT, without compromising tumour control [27,28,29,30,31]. Notably, a meta-analysis by Lin et al. [32] on the outcomes of IMRT use for the definitive treatment of cervical cancer included a total of 1008 patients (IMRT = 350, 3DCRT/2D-RT = 658) and showed a significant decrease in the incidence of acute GI and GU toxicities in patients treated with IMRT compared to 3DCRT/2D-RT. Odds ratios (OR) were 0.55 (95% CI: 0.32–0.95, *p* = 0.03 ) for grade ≥3 acute GI toxicities and 0.31 (95% CI: 0.14–0.67, *p*= 0.003) for acute GU toxicities [32]. Moreover, there were no difference in 3-year PFS and OS when comparing the two techniques [32], showing that IMRT confers equivalent efficacy compared to conventional techniques with a lower toxicity profile.

In the post-operative setting, RTOG 0418 was the first multi-institutional phase II study that evaluated the use of IMRT to the pelvis vs. four-field box conventional RT. The study included 83 patients, 43 with endometrial cancer and 40 with cervical cancer. Patients with endometrial cancer received IMRT alone, whereas patients with cervical cancer received IMRT and weekly cisplatin. The RT dose was 50.4 Gy in 28 fractions to the pelvic lymph nodes and vagina. An analysis of hematological toxicities from this cohort showed that patients with cervical cancer treated with IMRT had grades 1, 2 and 3 hematological toxicities of 23%, 33% and 25%, respectively and no grade 4–5 toxicities [33]. Importantly, the volume of bone marrow receiving 40 Gy correlated with acute hematological toxicities, as 75% of patients with a V40 > 37% experienced grade 2 or higher hematological toxicities compared to 40% of patients with a V40 ≤ 37% (*p* = 0.025). A median bone marrow dose of >34.2 Gy was also significantly associated with higher rates of grade ≥ 2 hematological toxicities; however, bone marrow is highly radiosensitive and keeping doses lower than 34.2 Gy is not sufficient to maintain hematopoiesis, as 43% of patients with a median bone marrow dose ≤34.2 Gy still experienced hematological toxicities [33]. The role of IMRT in the post-operative setting is equally advantageous to reduce acute GI toxicities, because with the uterus and ovaries removed, there is more room for the small bowel to shift downwards and occupy the pelvis, thus being more at risk of toxicity from RT. The TIME-C trial (NRG/RTOG 1203) is a phase III RCT that evaluated patient-reported acute GI toxicity from baseline to the end of RT, for 278 patients with cervical or endometrial cancer, randomized to post-operative RT using IMRT or a four-field box [34]. Secondary endpoints included a change in patient-reported urinary toxicity and quality of life. IMRT was associated with a significantly smaller decline in patient-reported bowel and urinary symptom scores, less frequent or constant diarrhea, and fewer use of anti-diarrhea medications [34].

#### 2.1.3. Adaptive External Beam Radiotherapy 

The uterus and cervix are highly susceptible to changes in position during RT delivery, mainly due to variations in bladder and rectal filling and tumour regression during RT [35]. The reported mean interfractional cervical motion varies between 2.3 and 16 mm in the anterior-posterior direction, 2.7 and 8 mm in the superior-inferior direction and between 0.3 and 10 mm in the lateral direction [36]. Thus, the concept of internal target volume (ITV) has been introduced to account for such variations in position [37]. The ITV is generated by doing simulation CT scans on a full and empty bladder and combining the CTV drawn on each of these scans to account for every position change between these two bladder-filling extremes. A PTV margin of 5–7 mm is then added to this ITV to account for setup and position in errors. However, cone beam CT (CBCT) is recommended so as to ensure adequate coverage of the CTV and PTV daily prior to RT delivery. Moreover, this approach has the benefit of decreasing the volume of OARs exposed to higher doses of RT and the use of image-guided IMRT has been associated with a decrease in GI and hematological toxicities compared with IMRT alone [38]. Other adaptive RT technologies have emerged over the past few years to further improve image guidance during EBRT delivery, notably the “plan of the day” approach or the “online adaptive RT” that include same day replanning and are the subject of a recently published review article [39]. 

### 2.2. Advances in Brachytherapy for the Treatment of Locally Advanced Cervical Cancer 

Brachytherapy (BT) is a type of RT in which small, sealed radioactive sources are placed in or near a tumour volume to deliver a therapeutic dose. It is an integral part of the treatment algorithm for LACC as it helps in boosting the RT dose to the local disease to a curative level. The addition of BT to the treatment of LACC is independently associated with a significantly higher survival rate, with up to a 12% absolute improvement in 4-year OS (from 46% to 58%, *p* < 0.001) with hazard ratios (HR) of 0.66 (95% CI, 0.60–0.74) [5]. In this SEER database analysis, BT was also associated with significantly improved cancer-specific survival (CSS) with a 4-year CSS of 64.3% vs. 51.5% for those who did not receive BT (*p* < 0.001) and HR of 0.64 (95% CI, 0.57–0.71). 

#### 2.2.1. From 2D-Brachytherapy (2D-BT) to 3D Image Guided Adaptive Brachytherapy (3D-IGABT)

Historically, BT for LACC was delivered through a 2D technique, whereby a 2 Gy equivalent cumulative dose (EQD2) of 80 to 85 Gy was delivered according to the Manchester system, to point A defined on X-ray. First introduced in 1938, the Manchester system defined point A as a point 2 cm superior to the external OS and 2 cm lateral and perpendicular to the applicator tandem, anatomically representing where the uterine vessels cross the ureter [40,41]. Thus, 2D-BT delivers a known dose to point A bilaterally, generating a pear-shaped distribution, but without factoring in patient-specific factors such as tumour size, anatomy, or doses to OARs, as these cannot be readily identified on an X-ray. However, with the advent of CT and MRI imaging during the procedure, the delivery of BT has evolved to a volume-based approach, taking into account variations in tumour size and position over the treatment course. This allows for conformal treatment of a high-risk clinical target volume (HR-CTV) while simultaneously sparing OARs.

**Figure 1:**
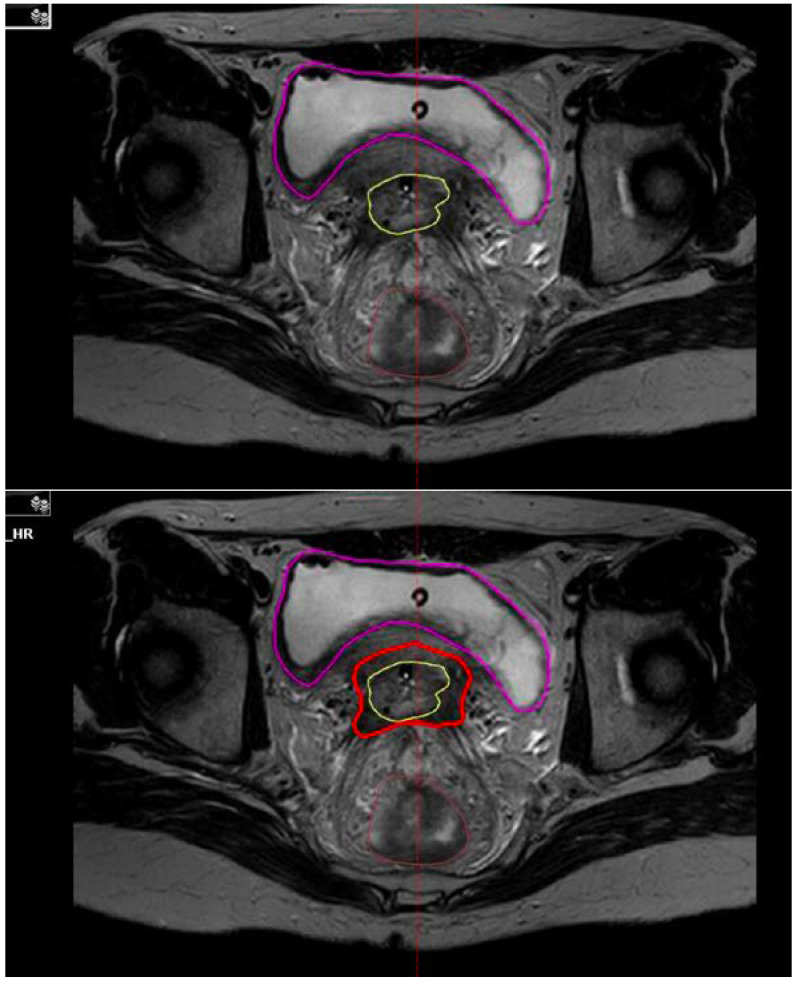
Residual gross tumour volume at brachytherapy (GTV-Tres: yellow) and adaptive High Risk CTV-T (CTV-T HRadapt: red) contoured at the time of brachytherapy on axial T2 MRI.

In 2005, the Groupe Européen de Curiethérapie and the European Society for Radiotherapy & Oncology (GEC-ESTRO) published the first recommendations on the basic concepts of terms utilized for a 3D image-based BT approach [42]. Since then, several consensus contouring guidelines and protocols have been developed for target delineation in cervical cancer brachytherapy [43,44,45]. Briefly, 3D-BT can be either CT-guided or MRI-guided, with the latter being considered the gold standard, as it is required for visualizing and contouring the residual GTV after EBRT. For instance, the EMBRACE-II protocol [46] defines the residual GTV (GTV-Tres) as the residual macroscopic tumour at the time of the BT boost, after treatment that is assumed sufficient to control microscopic disease, and based on clinical examination and imaging (MRI, PET/CT). The adaptive High Risk CTV-T (CTV-T HRadapt) includes the GTV-Tres, the whole cervix and adjacent residual pathologic tissue as defined by clinical examination and imaging at the time of BT (Figure 1). It is the volume bearing the highest risk for recurrence and should receive at least 90 Gy of cumulative EQD2 as per the EMBRACE-II dose targets (D90 CTV-T HR > 90–95 Gy EQD2_10_). The Intermediate Risk CTV-T (CTV-T IR) represents the area of the GTVinit superimposed on the topography at the time of brachytherapy and a margin surrounding the anatomical cervical borders in areas without an initial GTV-T. Dose distribution and optimization is achieved using different types of applicators that are MRI compatible. Intracavitary applicators can be used for smaller tumours (<31 cc) but are limited at covering the CTV-T HRadapt to doses >85 Gy, in case of larger tumours or asymmetrical local tumour extension for example to the parametria, vagina, bladder or rectal wall [47,48]. For such cases, a combined intracavitary-interstitial (IC/IS) approach is favoured and allows for improved conformality and target-dose escalation without increasing the doses received by the OARs [47,48,49,50]. 

#### 2.2.2. Outcomes of 3D Image-Guided Brachytherapy (3D-IGABT)

Several studies have reported improved LC and survival outcomes, and decreased toxicities with the use of 3D-IGABT compared to 2D-BT. Initiated in 2005, the French STIC trial was the first prospective, non-randomized trial to compare 2D- vs. 3D-BT in the treatment of LACC [51]. A total of 705 LACC patients were treated as per Group 1: BT (2D or 3D) followed by surgery; Group 2: chemoradiation, BT (2D or 3D) followed by surgery, or Group 3: chemoradiation then BT (2D or 3D), for which the 3D-BT was mostly CT-guided. At 24 months, improved LC was observed in all three groups, as well as a 50% reduction in grade 3 and 4 morbidity with 3D-BT compared to 2D-BT [51]. The RetroEMBRACE study is the largest retrospective, multi-institutional study of its kind reporting on the outcomes of 731 women with LACC, treated with CCRT and 3D-IGABT (either by CT or MRI) [52]. With a median follow-up of 43 months, Sturdza et al. reported excellent 5-year LC rates of 89%, pelvic control (PC) of 84%, cancer-specific survival (CSS) of 73% and OS of 65%, which is an improvement of ~10% compared to historic controls using 2D-BT. They also reported limited morbidity with grade ≥3 late toxicity rates of 5%, 7%, and 5%, respectively, for the bladder, gastrointestinal tract and vagina [52]. Importantly, for patients treated with MRI-based IGABT, 5-year LC was 94% for those with tumours <5 cm compared to 81% for tumours ≥5 cm (*p* ≤ 0.001), thus showing how tumour size impacts on LC. The recently published EMBRACE-I prospective, multicentre cohort study evaluated local tumour control and morbidity after chemoradiotherapy and MRI-based IGABT in LACC. They reported an actuarial 5-year LC of 92.0% (95% CI, 90–93) at a median follow-up of 51 months, 5-year PC of 87.0% (95% CI, 85–89) and 5-year nodal control of 87.0% (95% CI, 85–89). The 5-year disease-free survival (DFS) in this cohort was 68% (95%, CI 65–70) and 5-year OS was 74% (95%, CI 72–77) [53]. LC was similar across all of the stage groups; however there was a 14-17% absolute improvement in LC in FIGO stage IIIB disease compared to the RetroEmbrace cohort (92% LC vs. 75% in RetroEmbrace) [52,53]. This could be due to an increase in the use of MRI planning (100% vs. 19%) and interstitial needles (43% vs. 23%) in EMBRACE-I compared to RetroEmbrace. The initially reported late grade ≥3 bowel and bladder toxicity from the EMBRACE-I cohort were 5.9% and 5.3%, respectively, [54,55] but the most recent report showed an actuarial cumulative 5-year incidence of grade ≥3 GI toxicity of 8.5% (6.9–10.6), grade ≥3 GU toxicity of 6.8% (95% CI 5.4–8.6), 5.7% (4.3–7.6) vaginal toxicity and 3.2% (2.2–4.5) fistula events [53]. 

Taken together, these studies show the superior safety profile and efficacy, at least in terms of LC, of 3D-IGABT compared to 2D-BT techniques. This has been corroborated by a recent systematic review and meta-analysis by Kim et al. that reported a pooled hazard ratio (HR) for grade ≥3 toxicities of 0.54 for 3D-IGABT compared to 2D-BT (95% CI 0.37–0.77) [56]. There was also a significant improvement in locoregional recurrence-free survival (HR = 0.61; 95% CI, 0.40–0.93) and PFS (HR = 0.75; 95% CI, 0.59–0.96) favoring 3D-IGABT, but not in OS (HR = 0.65; 95% CI 0.40–1.06). Though MRI-guided IGABT is now considered the gold standard for brachytherapy boost in the treatment of LACC in most European and North American centres, the lack of clear OS benefit in this meta-analysis raises questions as to its widespread adoption, specifically in developing countries where access to MRI planning may be limited. However, the lack of clear OS benefit in this meta-analysis may be because 40% of patients in the pooled analysis were treated with CT-guided BT and only a few patients received interstitial BT, which could have negatively impacted OS rates. Moreover, a cost-effectiveness analysis performed in the US showed that 3D-IGABT is a cost-effective option compared 2D-BT, thus supporting its routine use in the treatment of LACC [57]. 

### 2.3. Stereotactic Body Radiotherapy in the Treatment of Locally Advanced Cervical Cancer

Stereotactic body radiotherapy (SBRT) is a type of EBRT whereby high doses of radiation per fraction (usually > 5Gy/fraction) are precisely delivered to a target in one or a few fractions. In recent years, SBRT has been considered as a conformal RT boost alternative to BT, particularly in patients unable to undergo BT due to unfavorable anatomy or medical comorbidities. A National Cancer Database registry analysis showed that from 2004 to 2011, the use of BT in LACC decreased from 96.7% to 86.1%, whereas the use of IMRT and SBRT as a radiation boost increased from 3.3% to 13.9% in the same period (*p* < 0.01). However, IMRT or SBRT boost were associated with inferior OS (HR = 1.86; 95% CI, 1.35-2.55; *p* <0.01), and this decrease was even more significant than that observed when excluding chemotherapy (HR = 1.61, 95% CI, 1.27–2.04, *p* < 0.01) [58]. Another National Cancer Database study later showed no significant difference in OS between the SBRT boost and brachytherapy boost after propensity score matching (HR = 1.477, 95% CI 0.746–2.926, *p* = 0.263), but there was a significant decrease in OS for patients who received IMRT boost vs. brachytherapy boost (HR = 1.455, 95% CI 1.300–1.628, *p* < 0.001) [59]. However, a recent single-arm phase II trial of SBRT boost (28 Gy in 4 fractions) as an alternative for intracavitary/interstitial BT boost for LACC was closed prematurely after 15 patients were enrolled, owing to concerns of toxicity [60]. Indeed, 2-year cumulative grade ≥ 3 toxicity was 26.7%, with predominantly rectal ulcers/fistulas. Two of the grade 3 patients died of complications from fistulas, resulting in a grade 5 toxicity rate of 13% [60]. Moreover, the efficacy of SBRT was inferior with 2-year LC, PFS, and OS of 70.1%, 46.7%, and 53.3%, respectively, although the authors argue that larger tumour size and patient comorbidities may have contributed to these inferior outcomes [60]. Another prospective study of SBRT boost for 25 gynecological patients with pelvic relapse or primary disease, of which seven were cervical cancer patients treated with definitive radiotherapy, reported a 1-year in-field RFS of 64.5% and 90.0% for the salvage and definitive group, respectively, and a 1-year OS of 80.8% and 49.1%, respectively [61]. One patient developed an entero-vaginal fistula, one developed sigmoid perforation and no patients experienced grade ≥ 3 genitourinary complications [61]. Taken together, it appears that SBRT boost in LACC results in inferior efficacy compared to BT, but more importantly, it can be associated with serious adverse effects. Thus, caution should be taken when considering this technique in LACC patients that cannot undergo brachytherapy and should only be attempted in the context of a clinical trial with special attention to the dose distribution to the bowel. In their dosimetric analysis, Albuquerque et al. showed that the percentage of rectal circumference receiving 15 Gy (PRC15) was associated with development of a grade 3 ulcer or rectovaginal fistula (*p* < 0.04), with PRC15 >62.7% being the strongest predictor of toxicity [60].

On the other hand, when SBRT is used for the treatment of oligometastic cervical cancer, it is associated with favorable response rates and LC. A meta-analysis of 17 studies on SBRT for oligometastic gynecological cancers, including 671 patients, 27.1% of which had cervical cancer, showed response rates ranging from 49% to 97% and LC ranging from 71% to 100% [62]. Disease progression occurred most commonly outside of the SBRT radiation field. Toxicity rates ranged from 2.6% to 10% and the majority of studies (9 out of 16 studies, 56%) did not report any grade ≥ 3 toxicities [62].

### 2.4. Immunotherapy as an Adjunct to Chemoradiation

Advances in chemoradiation for the treatment of LACC, notably the use of MRI-based IGABT, have translated into improved LC and toxicity profile. However, the OS for patients with advanced disease remains dismal, and this is thought to be mainly driven by distant failures rather than local recurrences. Thus, new systemic treatments are needed to improve OS for patients with LACC. One of such treatment adjuncts is immunotherapy. Indeed, cervical cancers are thought to be highly immunogenic, as a virus-driven type of cancer (HPV), thus amenable to respond to immunotherapy. Cervical cancer ranks amongst the tumours with the most somatic mutations, neoantigen formation and immune cell infiltrates [63,64]. Furthermore, a landmark Cancer Genome Atlas study on invasive cervical cancer identified several targetable mutations in this type of cancer, notably amplifications in the immune checkpoint regulators programmed death ligand (PD-L1 and PD-L2 [65]. Finally, several studies have shown that HPV positivity is associated with increased PD-L1 expression [66,67]. Taken together, all these factors argue for the rationale that cervical cancer tumours would respond to checkpoint-inhibitor targeted therapy. 

To date, a few studies have investigated the role of targeted anti PD1/PD-L1 therapy in cervical cancer. The Keynote-28 (NCT02054806) was a single-arm, phase IB basket trial of 477 patients from 20 different cohorts with advanced or metastatic PD-L1-expressing solid tumours, including 24 cervical cancer patients. Patients received pembrolizumab every two weeks for up to 24 months. The primary endpoint was overall response rate as per RECIST v1.1 criteria and secondary endpoint was safety. With a median follow-up of 11 months, the objective response rate (ORR) in the cervical cancer cohort was 17% (95% CI, 5% to 37%) with four patients achieving a partial response and median duration of response was 5.4 months (4.1 to 7.5 months) [68]. The 6-month PFS was 13% and 6-month OS was 66.7%. Treatment-related adverse events (AEs) were reported in 18 patients (75%) with five patients experiencing grade 3 treatment-related AEs. There were no grade 4 treatment-related AEs or deaths [68].

Furthermore, KEYNOTE-158 (NCT02628067) is an ongoing phase II trial including 1595 patients with advanced (unresectable and/or metastatic) solid tumours who have progressed to standard of care therapy and treated with pembrolizumab [69]. This included a total of 98 patients with previously treated advanced cervical cancer, of which 82 patients (83.7%) had PD-L1-positive tumours. Pembrolizumab monotherapy was administered every 3 weeks for 2 years until progression. The primary endpoint was ORR as per RECIST v1.1 criteria and secondary endpoints included PFS and OS. With a median follow-up of 10.2 months, ORR was 12.2% (95% CI, 6.5–20.4), with three complete and nine partial responses. All 12 responses were observed in patients with PD-L1-positive tumours. Median duration of response was not reached (range, ≥ 3.7 to ≥ 18.6 months) at time of interim analysis. Median OS was 9.4 months for the entire cohort and 11.0 months for the PD-L1-positive patients. Treatment-related AEs occurred in 65.3% of patients with grade 3 and 4 treatment-related AEs occurring in 12.2% of patients. The most common AEs were hypothyroidism (10.2%), decreased appetite (9.2%), and fatigue (9.2%) [69]. These two trials showed that pembrolizumab had durable anti-tumour activity in cervical cancer with acceptable toxicity. Based on this, the Food and Drug Administration (FDA) approved, in June 2018, the use of pembrolizumab for the second-line treatment of PD-L1-positive metastatic or recurrent cervical cancer. 

Nivolumab was also shown to have efficacy in advanced/metastatic cervical cancer in the second-line setting. In the phase I/II CHECKMATE 358 (NCT02488759), 24 patients with recurrent or metastatic gynecological cancers (19 cervical, 5 vaginal/vulvar) who had received no more than two previous lines of treatment, were treated with nivolumab every two weeks regardless of PD-L1 status [70]. With a median follow-up of 19.2 months, ORRs were 26.3% (95% CI, 9.1–51.2) for cervical cancer and 20.0% (95% CI, 0.5–71.6) for vaginal/vulvar cancers. The median duration of response (DOR) was not reached (range, 23.3 to 29.5+ months) at the time of analysis in the five responding patients in the cervical cohort. Median OS was 21.9 months (95% CI, 15.1–not reached) for cervical cancer patients. Treatment-related AEs were reported in 12/19 patients (63.2%) in the cervical cohort and all five patients in the vaginal/vulvar cohort, with 21.1% (*n* = 4) being of grade 3 and 4 [70].

In June 2021, Merck announced that a Phase 3 double-blind, randomized trial KEYNOTE-826 (NCT03635567) met its primary endpoint of OS and PFS for the first-line treatment of patients with persistent, recurrent or metastatic cervical cancer. A total of 548 patients with a PD-L1 combined positive score of 1 or more received in a 1:1 ratio pembrolizumab or placebo every 3 weeks plus platinum-based chemotherapy and, per investigator discretion, bevacizumab. Primary endpoints were PFS and OS. The results of the first interim analysis were just published in the November 2021 edition of the New England Journal of Medicine, and showed a median PFS of 10.4 months in the pembrolizumab group and 8.2 months in the placebo group (HR for disease progression or death of 0.62; 95% CI, 0.50–0.77; *p* < 0.001) [71]. The 2 year-OS was 53.0% in the pembrolizumab group and 41.7% in the placebo group (HR for death of 0.64; 95% CI, 0.50–0.81; *p* < 0.001) [71]. Grade 3 to 5 AEs occurred in 81.8% of patients in the pembrolizumab group and in 75.1% of patients in the placebo group, with grade 5 events occurring in 14 patients in each group (4.6% and 4.5%, respectively) [71].

In the definitive setting, ENGOT-cx11/KEYNOTE-A18 (NCT04221945) is a phase III, randomized trial evaluating the combination of pembrolizumab with concurrent CRT for the treatment of locally advanced cervical cancer [72]. It is still ongoing and aims to recruit 980 patients with high-risk LACC (FIGO 2014 stage IB2-IIB with node-positive disease or stage III-IVA) who have not received prior treatments, randomized 1:1 to receive either 5 cycles of pembrolizumab vs. placebo every 3 weeks plus CRT followed by 15 cycles of pembrolizumab vs. placebo every 6 weeks. The CRT regimen is as per the standard practice, including 5–6 cycles of cisplatin 40 mg/m^2^ weekly + EBRT followed by brachytherapy (IGABT). Randomization will be stratified based on the EBRT technique (IMRT or VMAT vs. non-IMRT), cancer stage at screening (stage IB2-IIB vs. III-IVA) and planned total RT dose. The primary endpoints are PFS as per RECIST v1.1 and OS. The secondary endpoints are 2-year PFS, 3-year OS, complete response at 12 weeks, ORR, PFS and OS in PD-L1–positive patients, EORTC QLQ-C30 and QLQ-CX24, and safety. Results of this trial are highly anticipated and will further elucidate whether immunotherapy combined with definitive CRT can improve LC, PC and survival in patients with LACC. Currently, there are ten clinical trials assessing the combination of immunotherapy and definitive chemoradiation in the treatment of cervical cancer (Table 1). Results from all these trials are eagerly awaited to assess whether immunotherapy could improve distant control as well as survival rates in LACC without significantly increasing toxicities.

## 3. Conclusions and Perspectives

RT plays a primordial role in the treatment of LACC. Radiation oncology technologies have progressed rapidly in the past two decades. Notably, the use of IG-IMRT and 3D-IGABT have considerably improved treatment outcomes and toxicity profiles for patients with LACC and are now considered the gold standard in many countries. However, there is still room for improvement, and new experimental perspectives include the addition of immunotherapy to chemoradiation regimens, or a move towards an even more personalized approach to treatment with the identification of risk factors and biomarkers that can be used to de-escalate or intensify treatments according to individual patients’ risk group (EMBRACE III). Other technological innovations such as the use of the SBRT boost to replace BT boost have been associated with increased toxicity and decreased efficacy and so should be used with caution and only in the context of clinical trials.

## Figures and Tables

**Table 1:** Clinical trials combining immunotherapy with definitive radiotherapy in the treatment of locally advanced cervical cancer.

Trial ID	Design	Eligibility	Intervention	Details	Outcome Measures	Status
NCT04221945 (KEYNOTE-A18/ENGOT-cx11/GOG-3047)	Randomized Phase III	FIGO 2014 Stage IB2–IIB (with N+ disease) or FIGO 2014 Stages III–IVA cervical cancer	**Pembrolizumab** + CRT + BT vs. Placebo + CRT + BT	Pembrolizumab 200 mg IV vs. placebo q3 weeks × 5 cycles, followed by Pembrolizumab 400 mg IV vs. placebo q6 weeks × 15 cycles + Cisplatin qweek during EBRT + BT (to a total RT dose of 80 Gy for volume directed and 75 Gy for point directed	Primary: PFS (RECIST 1.1), OS Secondary: 2-year PFS, 3-year OS, CR at 12 weeks, ORR, PFS and OS in PD-L1+ patients, PFS after next line treatment, EORTC QLQ-C30, QLQ-CX24, and safety	Recruiting
NCT02635360	Randomized Phase II	Confirmed cervical Cancer (excluded: distant metastases)	**Pembrolizumab** following CRT vs. Pembrolizumab concurrent with CRT	CRT followed by Pembrolizumab 200 mg IV q21 days × 3 months Vs Pembrolizumab 200 mg IV q21 days at the same time as CRT	Primary: Change in immunologic markers, Incidence of DLTs Secondary: Metabolic Response Rate on PET/CT, Incidence of distant metastases, PFS, OS	Active, not recruiting
NCT03738228	Multi-arm Phase I	Stage IB2, II, IIIB, or IVA cervical cancer	**Atezolizumab** + CRT + BT	Arm A: Atezolizumab IV on **days -21, 0, and 21** + Cisplatin qweek concurrent with EBRT (Monday–Friday) × 5 weeks + IGBT at week 4 or 5 Arm B: Atezolizumab IV on **days -21, 0, and 42** + Cisplatin qweek concurrent with EBRT (Monday-Friday) × 5 weeks + IGBT at week 4 or 5	Primary: T cell receptor beta (TCRB) clonal expansion in peripheral blood Secondary: Incidence of DLTs, Frequency and severity of AEs as per CTCAE v5, TCR clonality, diversity, and frequency in peripheral blood and tissue, PD-L1 expression in tissue	Active, not recruiting
NCT03612791 (ATEZOLACC)	Randomized Phase II	FIGO 2009 stage IB1–IIA (N+) or stage IIB–IVA cervical cancer	**Atezolizumab** + SoC CRT + BT vs. SoC CRT + BT	Atezolizumab 1200 mg IV q3 week starting on week1 and continued as adjuvant treatment for a max of 20 cycles + Cisplatin qweek concurrent with pelvic +/− para-aortic EBRT by IMRT (45Gy/25Fx) + BT starting at week 7 (85 Gy EQD2 to HR-CTV) vs concurrent CRT +BT alone as above	Primary: PFS (RECIST 1.1)	Recruiting
NCT03527264 (BrUOG 355)	Non-randomized Phase II	Cervical cancer	**Nivolumab** induction + Nivolumab concurrent with chemoradiation + Nivolumab maintenance	Cohort 1A: Nivolumab induction (240 mg IV × 2 doses) + Nivolumab 240 mg IV q14 days for 3 doses concurrent on day 1 with Cisplatin qweek and EBRT (45 Gy/25 Fx) Cohort 1B: As above but with EFRT Cohort 2: Nivolumab induction as above + CRT w/o Nivolumba + Maintenance Nivolumab (480 mg IV q4weeks × 2 years) Cohort 3: Nivolumab induction + Nivolumab with CRT + Maintenance Nivolumab	Primary: Feasibility of the incorporation of nivolumab with weekly cisplatin and EFRT or WPRT in women with cervical cancer (acute toxicities as per CTCAE v4.0), PFS	Active, not recruiting
NCT03298893 (NiCOL)	Single arm Phase I/II	Stage IB2–IVA squamous-cell carcinoma or adenocarcinoma of the cervix	**Nivolumab** + CRT followed by 5 months of Nivolumab alone	Nivolumab IV q2 weeks + Cisplastin + EBRT (45Gy/25Fx by IMRT/VMAT +/− SIB to 54Gy/25Fx)	Primary: rate of DLT Secondary: ORR, PFS, DFS, Incidence of SAEs and AEs, molecular alterations, ctDNA heterogeneity, tumour microenvironment description, tumour PD-L1 IHC	Active, not recruiting
NCT03830866 (CALLA)	Phase III RCT	FIGO (2009) Stages IB2 to IIB N+ or FIGO (2009) IIIA–IVA any node cervical adenoCa or SCC	**Durvalumab** + SoC CRT + BT followed by Durvalumab monotherapy up to 24 months or until progression of disease, vs. Placebo + SoC CRT + BT	Durvalumab IV q4 weeks + Cisplatin (or Carboplatin) qweek concurrent with EBRT + BT	Primary: PFS (RECIST 1.1) Secondary: OS, CR (RECIST 1.1), duration of response, QoL (EORTC QLQ-C30, EORTC CX24), 3-year PFS, PFS and OS in PD-L1+ patients	Active, not recruiting
NCT01711515	Single arm Phase I	Stage IB2–IIA with positive PA LNs, IIB/IIB/IVA with positive pelvic or PA LNs cervical cancer	CRT + BT+ adjuvant **Ipilimumab**	Cisplatin qweek + EBRT × 6 weeks + BT followed by Ipilimumab IV q3weeks for 12 weeks	Primary: DLTs occurring during adjuvant ipilimumab in the dose escalation phase, DLTs occurring in the feasibility phase, AEs Secondary: Response rate (RECIST 1.1), PFS, OS, location of recurrence (locoregional versus distant), chronic toxicities	Completed
NCT01158248	Phase II	Stage IB–IIIB cervical cancer with no PA LNs	**Panitumumab** + CRT + BT	Panitumumab + CRT+ BT	Primary: PFS at 4 months by MRI according to RECIST, Rate of skin and/or gastrointestinal toxicity CTCAE grade 4 at 4 months Secondary: ORR at 4 months according to RECIST criteria, PFS and OS at 12 and 24 months, rate of SAEs at 4, 12, 24 months, Rate of SAEs of panitumumab monotherapy at day 14	Unknown
NCT04580771 (IMMUNOCERV)	Single arm Phase II	Stage IB3–IVA cervical cancer	**Liposomal HPV-16 E6/E7 Multipeptide Vaccine** (PDS0101) + SoC CRT (Cisplatin + RT)	RT (Monday–Friday) for 5–7 weeks _ Cisplatin IV qweek during the 5 weeks of RT + PDS0101 SC on days -10, 7, 28, 49, and 170 in the absence of disease progression or unacceptable toxicity.	Primary: Rate of grade ≥ 3 acute toxicity Secondary: complete metabolic response rate of ≥ 90% GTV reduction, LC, PFS, OS at 12 and 18 months, Long term safety (rate of grade ≥3 chronic toxicity)	Recruiting

RT: radiotherapy; EBRT: external beam radiotherapy; IMRT: intensity modulated radiotherapy; BT: brachytherapy; SoC CRT: standard of care chemoradiation consisting of weekly cisplatin concurrent with pelvic +/− para-aortic EBRT (45Gy/25Fx) followed by BT (to 80–90 Gy EQD2); EFRT: extended field radiotherapy, WPRT: whole pelvic radiation therapy; N+: node positive, LNs: lymph nodes, PA: para-aortic, adenoCa: adenocarcinoma; SCC: squamous cell carcinoma; ORR: objective response rate; PFS: progression-free survival; OS: overall survival; DFS: Disease Free Survival; MTD: maximum tolerated dose; dose-limiting toxicities (DLTs); AEs: adverse events; SAEs: serious adverse events.
